# Wealth comparison across social distances: implications for well-being

**DOI:** 10.3389/fpsyg.2025.1661009

**Published:** 2025-11-26

**Authors:** Yingying Wu, Qinglan Tang, Zhongli Huang, Ziyu Liu, Kayan Liang

**Affiliations:** 1 Department of Business, University of Nottingham Ningbo China, Ningbo, China; 2 Reading Academy, Nanjing University of Information Science and Technology, Nanjing, China; 3 International College, China Agricultural University, Beijing, China; 4 Law School, Sun Yat-sen University, Guangzhou, China; 5 School of Business, Sun Yat-sen University, Guangzhou, China

**Keywords:** wealth comparison, well-being, social distances, stress, help-seeking behavior

## Abstract

**Introduction:**

In today’s digitally connected and economically unequal world, upward wealth comparisons are pervasive. This study examined how wealth comparisons across different social distances (family, friends, and internet) distinctly affect well-being.

**Methods:**

We employed a scenario-based questionnaire design to assess the effects of wealth comparisons. Data were analyzed using regression models, with stress tested as a mediator through mediation analysis and heterogeneous effects explored across subgroups based on help-seeking behaviors.

**Results:**

Comparisons with all three groups are associated with negative influence on well-being, with comparisons to friends exhibiting the strongest effect. Stress mediates these impacts, while help-seeking behaviors show divergent pathways. Additionally, life satisfaction and income buffer sensitivity to disparities.

**Discussion:**

The findings underscore that the risk of upward wealth comparisons is contingent on social distance. This research integrates offline and online dynamics into a cohesive theoretical framework, advancing social comparison theory and providing actionable insights for interventions designed to protect well-being in the face of pervasive social comparison.

## 1 Introduction

Recent global surveys indicate a marked decline in self-reported happiness, with younger generations experiencing the most significant reductions in well-being. According to the Ipsos Global Happiness Report (Ipsos News, 2024), only 65% of Generation Z feel in control of their lives, compared to 76% of Baby Boomers. Gen Z also reports lower satisfaction with their mental health (63%). This generational gap in well-being highlights a question to understand the psychological and social mechanisms that may undermine well-being among young people. Sirgy (2019) mentions that individual perceptions of quality of life comes from a comprehensive assessment of satisfaction with multiple life domain (e.g., health, wealth, and social). Lyubomirsky (2001) contend that sustained differences in happiness from stable cognitive-motivational patterns and intentional activities—among which social comparison plays a central role.

Against this backdrop of declining happiness, social comparison emerges as a particularly relevant mechanism. Social comparison, a fundamental process of self-evaluation through comparisons with others, has been shown to significantly reduce subjective well-being (Festinger, 1954). In particular, upward social comparison—comparing oneself with others who are better off—is associated with emotions such as envy, resentment, and reduced self-esteem, thereby linking to lower hedonic well-being (White et al., 2006). Hedonic well-being, which comprises cognitive life satisfaction and the balance between positive and negative affect (Disabato et al., 2016), is especially vulnerable to upward comparisons involving salient attributes such as wealth, which prompt strong emotional and evaluative reactions.

While downward comparisons (comparing oneself to worse-off others) are theorized to boost well-being through enhanced self-esteem (Wills, 1981), contemporary research underscores its limited and potentially detrimental role. Research shows that such comparisons can elicit anxiety about one’s own social and economic vulnerability (Lockwood, 2002), as well as provoke guilt or empathetic distress that counteracts any self-enhancement benefits (Exline and Lobel, 1999). Furthermore, in an era of pervasive digital media and rising inequality, upward comparisons tend to dominate social cognition and exert a stronger influence on well-being than downward comparisons (Cheung and Lucas, 2016). Owing to their heightened salience and emotional intensity—especially on visible dimensions like—upward wealth comparisons serve as a key mechanism through which perceptions of relative deprivation and symbolic inferiority undermine hedonic well-being (Collins, 1996; Salovey and Rodin, 1984). Accordingly, this study focuses primarily on upward social comparison to elucidate its role in influencing well-being in contemporary societal contexts.

The mechanism through which wealth comparisons impair well-being is multifaceted (Oishi et al., 2022). Wealth comparisons trigger stress evaluations, which mediate the pathway from comparative cognition to well-being impairment. Transactional model of stress views stress as a dynamic process of an individual’s interaction with the environment, emphasizing that cognitive assessment triggers stress responses, which in turn impairs well-being (Lazarus et al., 1984). Complementarily, the stress-buffering hypothesis (Cohen and Wills, 1985) proposes that social support affects the negative effects of stress on well-being and seeking help as the main form of social support is an important factor influencing the reduction of well-being caused by wealth comparison. The local dominance effect (Zell and Alicke, 2010) and psychological distance theory (Trope and Liberman, 2010) postulate that perceptions of relational proximity elicit distinct emotional responses. Siedlecki et al. (2014) identify emotional bonds as critical moderators of social comparison outcomes, while help-seeking behavior may act as a catalyst or buffer for declining well-being.

Building on these models, this study proposes that upward wealth comparison is related to a reduction in hedonic well-being, but is influenced by stress and help-seeking behavior. Current literature, however, often isolates online and offline environments or conflates comparison targets and has predominantly focused on comparisons with familiar individuals in offline contexts [e.g., the neighbor effect of ‘Keeping up with the Joneses’ (Guven and Sørensen, 2012)], which constrains the generalizability of findings to contemporary digital and urban contexts. It is suggested that urbanization and digitalization are progressively reducing the relevance of neighbors as primary reference groups for social comparison, particularly within major metropolitan areas worldwide (Putnam, 2020). Within this transforming social fabric, familial bonds continue to constitute a foundational relational context for comparison in many cultural settings (Tesser et al., 1988), while friendships remain a persistent and salient source of everyday social evaluation (Festinger, 1954). Concurrently, digital platforms have evolved into a ubiquitous arena for social comparison, enabling individuals to make lateral and upward comparisons that transcend geographical, social, and cultural boundaries (Vogel et al., 2014). In response to these macro-social shifts, this study explicitly categorizes social comparison targets into three distinct tiers of social distance: family, friends, and internet.

Family comparisons carry unique well-being implications, blending protective and detrimental outcomes. The convoy model of social relations posits that family members typically populate the inner circle of one’s support network, exerting substantial influence on well-being through supportive and conflictual exchanges (Webster et al., 2022). While familial relationships offer stability and emotional security, they simultaneously serve as potent comparison benchmarks, particularly in the existence of expectation or resource disparities (Fingerman et al., 2020). Notably, family support plays a dual role—enhancing life satisfaction when aligned with individual needs but potentially amplifying negative affect when perceived as insufficient or controlling (Siedlecki et al., 2014). Moreover, individuals suppress help-seeking behaviors to avoid perceived burdensomeness or relational disharmony, leading to the persistence of negative emotions (Taylor et al., 2004).

Friendships function not only as contexts for comparison but also as sources of emotional protection and resilience. The local dominance effect posits that individuals exhibit heightened sensitivity to psychologically proximal targets, rendering friends particularly salient comparison targets (Zell and Alicke, 2010). Additionally, the valence of such comparisons is moderated by relational quality: high-quality friendships characterized by mutual support and reciprocity can reframe upward comparisons as inspirational rather than threatening, thereby mitigating their detrimental effects (Siedlecki et al., 2014; Collins and Feeney, 2004). Corroborating this, Taylor et al., 2004 find that friendship closeness enhances well-being and self-esteem while buffering the stressful effect of other relationships. Particularly, this buffering function can not only cushion the negative emotions of comparison by offering a direct emotional scaffold, such as comforting behaviors, but can provide an alternative perspective to attenuate or counteract the effects of family conflict – even under family stress, individuals with close friendships ties report sustained high well-being (Uchino, 2009).

Social media serves as a powerful platform that universally and directly stimulates the generation of social comparisons and influences well-being. Unlike real-world interactions, algorithmically curated networks amplify exposure to strangers’ lifestyles, facilitating upward comparisons (Amichai-Hamburger and Vinitzky, 2010). Distinct from family or friends, interactions on digital platforms are well-planned and purposeful, triggering unrealistic comparisons (e.g., luxury displays) that exacerbate frustration and dissatisfaction (Vogel et al., 2014). This phenomenon aligns with “Happiness Paradox” where individuals perceive their social media peers as happier due to the structural biases of online networks (Bollen et al., 2017). Furthermore, by distinguishing between passive and active platform use, it finds that passive browsing elicits envy, as unidirectional consumption of curated wealth displays (e.g., vacations) reduces self-esteem through comparisons (Verduyn et al., 2017) and these effects can be exacerbated by the widening wealth gap (Oishi et al., 2022; Oishi et al., 2011).

Based on the above analysis, this paper proposes a hypothesis as below.

*H1*: The effect of upward wealth comparison on well-being is negatively affected by family, friends and internet respectively.

*H2*: Stress plays a mediating role in the relationship between wealth comparison and well-being.

*H3*: Help-seeking behavior regulates the relationship between stress and well-being.

To explore this hypothesis, this study adopts a scenario-building approach, collecting data by through questionnaire distribution, and employs regression analysis to investigate how wealth comparisons across these dimensions differentially affect well-being. It further examines the mediating role of stress and the heterogeneous effects of help-seeking behavior. Additionally, another regression model is created based on scenarios with varying wealth gaps to identify the drivers of well-being changes across social distance groups.

Results indicate that social comparison exerts a negative effect on well-being, most pronounced in the friends group. Stress and help-seeking behaviors mediated the comparison adverse effects, with the primary drivers of well-being variation being life satisfaction and income. These findings aim to advance theoretical models of social comparison and provide practical insights for interventions to mitigate comparison-driven loss of well-being in an increasingly stratified world.

This paper contributes to literature as follows. Firstly, we propose a tripartite social distance framework instead of the online-offline dichotomy, better reflecting the reality of internet integration into daily life. Secondly, introducing help-seeking behavior, discussing its buffering and catalytic effects separately, explaining the paradoxes of some models in modern times and enriching the theory. Thirdly, developing a scenario-based methodology to quantify wealth gap effects, enabling targeted well-being interventions.

## 2 Methodology

### 2.1 Survey and sample

Our data collection is based on a laboratory experiment of online survey. We collect data through questionnaires, which can capture the immediate psychological changes caused by the comparison situation to obtain relatively accurate results. We design an online survey with consideration of data availability while ensuring operationalization of core variables—including social distance, wealth gap, stress, and help-seeking behavior. In this survey, we include two parts: basic information and scenarios for treatment effect. We send three separate questionnaires, and each questionnaire includes Scenario 1 and targeted Scenario 2. Each questionnaire includes three sections.

Section 1 is basic information collection. Participants provide basic information, including age, gender, annual income, educational background, occupation, social engagement level, lifestyle satisfaction, stress in daily life, self-esteem, health status, relative well-being at income level, relative well-being at diet and seek help. Additional targeted questions are based on different groups, for instance, the family group questionnaires include living with family, family gatherings and trips, communication with family and family harmony.

In section 2, we construct scenarios. The questionnaire targets family, friends, and internet, and designs everyday scenarios including “house gatherings,” “class reunions,” and “online browsing.” The wealth disparity is articulated across five key dimensions: income, work, assets, consumption, and leisure. Concrete numerical values (e.g., ¥8,000 per month) serve as cognitive anchors that enhance the tangibility of the comparison (Tversky and Kahneman, 1974).

Scenario 1 reflects the profile of China’s middle-class wealth as described in theories from institutions such as CPCNews (Li, 2017). In contrast, Scenario 2 depicts a significantly elevated socioeconomic status across all dimensions. Furthermore, specific symbolic cues such as reference to a Porsche 911 or exclusive jewelry exhibitions are incorporated based on insights from Bain & Company (Bain and Company, 2024). These elements activate pre-existing cognitive and emotional associations with luxury and success, thereby strengthening participants’ perception of scenario 2 (Smith and Kim, 2007).

After the scenarios, we collected dependent variables (well-being, satisfaction, stress in the scenario, meaning, and change) in Section 3 using a single-item 0–10 scale. This efficient approach was adopted to capture immediate responses and minimize cognitive burden, which is supported by its strong correlation with multi-item scales (Abdel-Khalek, 2006). Finally, we set the question: “How much do you think this life is true?” to help measure the participants’ perception of the realism of scenarios. All specific questions in scenarios shows in Table A-1.

The questionnaires were collected online from 15th January to 30 January to Credamo and WeChat platform, with a total of 324 responses collected. This questionnaire strives to clarify the formulation of scale items, avoid complex sentence patterns or double meanings, and clearly emphasize the confidentiality and anonymity of data to respondents, and encourage more realistic answers. After screening for abnormal questionnaires including missing information and unclear answers (such as “anything is OK”), the actual number of valid responses is 303.

As each participate is engaged in two scenarios with random sequence, we treat each respondent as participating the survey for two times and generate 606 observations. The sample is collected from family group, friend group, and internet group according to the difference of social distance and are analyzed as main independent variables. To identify possible internal driving factors, we conducted differential analysis using 303 data with Scenario 1 as the control group and Scenario 2 as the experimental group (Table 1).

**Table 1:** Scenario design.

Scenario	Scenario 1: a modest lifestyle	Scenario 2: high-level lifestyle
	Hypothetical life status for participants	At a family gathering, cousin’s life situation At the class reunion, a classmate’s life situation While browsing the web, someone sharing his life
Income	Monthly income: ¥8,000, no debt.	Annual income: ¥2million, supplemented by investments and financial portfolios.
Work	Fixed working hours with attendance requirements.	Flexible, self-directed work schedules.
Property	Ownership of a 90 m^2^ standard apartment.	Ownership of a 300 m^2^ luxury urban residence and a suburban villa.
Consumption	Clothing normally less than ¥500, a ¥5,000 smartphone use 2 years.	Consumption of luxury brands for clothing and the latest electronic devices.
	Daily meals averaging ¥20 per person.	Frequent high-end dining (¥500 + per person).
	A ¥100,000 electric car.	A Porsche 911 (valued at ~¥1.5 million).
Entertainment	Primary use of public transportation; two domestic trips annually; leisure activities in public spaces (e.g., parks).	More than international trips annually; participation in exclusive events (e.g., jewelry exhibitions); expensive hobbies (e.g., skiing).

### 2.2 Variables

Our main dependent variable is well-being, measured on a scale from 0 to 10. We also provide alternative dependent variable, satisfaction.

Our independent variables are comparisons. We define Family group as a dummy variable, which is equals 1 if the participant is involved in the questionnaire of family group, otherwise equals 0. The same applies to friend group and internet group.

Control variables include age, age square, gender, ln(income), education, hobbies, occupation, social engagement, life satisfaction, stress in daily life, relative well-being at income level, relative well-being at diet, health, self-esteem, seek help family (dummy), and seek help friend (dummy). The variable “diverse hobbies” is defined as 1 if the number of hobbies exceeds three, derived from the survey question: “How many hobbies do you have?”. Life satisfaction is measured on a 0–10 scale based on the question: “How satisfied are you with your current life?”, where 0 denotes “completely dissatisfied” and 10 denotes “very satisfied.” Stress in daily life is also measured on a scale from 0 to 10, with 0 representing “no stress” and 10 indicating “extremely high stress.” Help-seeking behavior is represented by two dummy variables: seeking help family (if prefers turning to family equals 1) and seeking help internet (if prefers turning to internet equals 1). Ln(income) is log-transformed income (¥0000, per year). Detailed descriptions of all control variables can be found in Table A-2.

In addition, we use the Harman one-factor test to assess the severity of common method bias (CMB) (Podsakoff et al., 2024), which shows that common method bias interfered with the results of this study within acceptable limits (Table A-3).

From the descriptive statistics in Table 2, education (M = 0.851, SD = 0.356) indicates that the respondents have a high level of education. The mean of stress in daily life is 6.092, and the standard deviation is 2.461, indicating that the current stress is high with large variation. Descriptive statistics on all variables for the three treatment groups can be found in Table A-4.

**Table 2:** Summary Statistics.

Variable	Mean	Standard deviation	Minimum	Maximum
*All sample (N = 303)*				
Age	28.102	10.049	17	65
Gender	0.502	0.500	0	1
Education	0.851	0.356	0	1
Income	8.752	10.258	1.2	100
Occupation	1.116	1.156	0	3
Health	0.165	0.371	0	1
Diverse hobbies	0.865	0.342	0	1
Social engagement	6.667	1.783	2	10
Life satisfaction	7.347	1.730	1	10
Stress in daily life	6.092	2.461	0	10
Self-esteem	7.693	1.780	1	10
Relative well-being at income level	8.172	1.730	0	10
Relative well-being at diet	7.168	1.964	0	10
Seek help family (D)	0.554	0.498	0	1
Seek help internet (D)	0.119	0.324	0	1

Dummy variables are denoted as D in parentheses.

We conduct a correlation matrix for the main variables, and the results are shown in Table A-5. To diagnose multicollinearity, we perform variance inflation factor (VIF) tests. While age and age square exhibit expectedly high collinearity, this exerts negligible impact on the core explanatory variables. In addition, the minimum VIF value of the other variables is 1.12 and the maximum value is 2.50 (Table A-6), indicating that the collinearity results meet the relevant test criteria, which confirms that there is no multicollinearity problem in the independent variables selected in this paper.

In summary, the variables are appropriately measured and constructed, and the empirical specifications satisfy the necessary diagnostic requirements for regression analysis.

### 2.3 Model

We construct a model investigating relationship between well-being and three group of social comparison in Equation 1:


$$Wellbeing_{i}=\beta T_{i}+\gamma X_{i}+u_{i}$$ (1)

where Wellbeing is personal’s well-being, the treatment variables in $T_{i}$ are family, friend, and internet, β is the coefficient showing the relationship, $X_{i}$ is the vector of controls, and $u_{i}$ is the error term. We estimate this equation using OLS. We also introduce Ordered logit model to test the robustness of estimation.

## 3 Empirical findings

### 3.1 Baseline regression and robustness check

Table 3 reports the relationship between wealth comparison and well-being. Column (1) shows that all comparisons (with family, friend, and internet groups) have statistically significant negative effects on well-being comparing with control group. Comparison with friends has the biggest adverse effect on well-being.

**Table 3:** Regression Results and Robustness Check.

	(1) Baseline without variables	(2) Baseline	(3) Alternative dependent variable	(4) Subsample analysis	(5) Alternative model
Dependent variable	Well-being	Well-being	Satisfaction	Well-being	Well-being
Method	OLS (1)	OLS (2)	OLS (3)	OLS (4)	Ordered Logit
Family group	−1.013***	−1.053***	−2.302***	−1.110***	−1.063***
	(0.214)	(0.202)	(0.211)	(0.225)	(0.215)
Friend group	−1.372***	−1.358***	−2.382***	−1.365***	−1.315***
	(0.209)	(0.196)	(0.206)	(0.214)	(0.211)
Internet group	−1.013***	−0.988***	−1.756***	−1.001***	−1.072***
	(0.216)	(0.204)	(0.214)	(0.226)	(0.215)
Diverse hobbies	All	All	All	Hobbies = 1	All
Control variables	No	Yes	Yes	Yes	No
Observations	606	606	606	524	606
R-squared	0.090	0.228	0.344	0.218	-

The significance level is denoted: *** for *p* < 0.01. Control variables include age, age square, gender, ln(income), education, occupation, social engagement, life satisfaction, stress in daily life, relative well-being at income level, relative well-being at diet, health, self-esteem, seek help family and seek help internet. Diverse hobbies = 1 if individual has more than three hobbies.

Comparing columns (1) and (2), the coefficients for the three treatments change show only slight changes while retaining high statistical significance (all *p*-values: 0.000). The differences between treatments become more pronounced. Social comparison of wealth is associated with reduction in well-being, and the magnitude of this negative effect varies with social distance: it is most detrimental for comparisons with friends (*β* = −1.358), followed by family (*β* = −1.053), and least for comparisons with internet groups (*β* = −0.988). Specifically, relative to the baseline group with no social comparison, well-being decreases by 1.358 units (0–10 scale) when comparing with friends—a reduction that is 0.305 units larger than that associated with family comparisons and 0.370 units larger than that associated with internet comparisons. This finding aligns with and extends the Local Dominance Effect (Zell and Alicke, 2010), demonstrating that friends, as psychologically proximate peers, constitute the most salient and impactful reference group for upward social comparison, which is associated with the strongest negative influence on well-being.

To assess the robustness of these baseline findings, we conduct three supplementary analyses.

First, we employ an alternative dependent variable. Following the definition of hedonic well-being (Disabato et al., 2016), we use responses to satisfaction (e.g., ‘Overall, how satisfied is your life in this scenario?’, choose from a range of 0–10) in questionnaires as a proxy for subjective well-being in robustness checks, which correlated with the original well-being index of 67.0% (Table A-5). Column (3) demonstrates that both the coefficient magnitudes and statistical significance remain consistent across treatment groups (family, friend, Internet), with friend comparisons consistently showing the strongest adverse effect.

Second, we examine a specific subsample. While the control variable “diverse hobbies” is not statistically significant, we test the robustness of our findings within the subsample of individuals reporting “Diverse hobbies = 1” (*N* = 524). As shown in column (4), social comparisons are persistently and significantly associated with lower well-being, with comparisons in friends exhibiting the strongest adverse effect.

Third, in column (5), we also introduce Ordered logit model to investigate the impact of comparisons in three groups on well-being, which shows a consistent and robust results with column (2). These robustness checks collectively reinforce the reliability and consistency of the baseline findings reported in column (2).

### 3.2 Mediation effect of stress

This section investigates the mediating role of stress in the relationship between social comparison and well-being across social distance. We employ a dual analytical approach: Baron and Kenny’s (Baron and Kenny, 1986) causal steps method to establish preliminary evidence of mediation through linear regression, and bias-corrected bootstrap sampling to quantify the indirect effect and address potential non-normality in the data (Preacher and Hayes, 2008).

Table 4 presents the three-step mediation results. Column 1 (the baseline regression) shows a significant association between social comparison and lower well-being, with comparisons among friends exhibiting the strongest negative correlation. In Column 2, where stress serves as the dependent variable, all three social comparison groups show significant positive associations with stress, satisfying the second condition for potential mediation. The coefficients indicate that social comparisons are substantially correlated with increased stress levels, with differences among groups being relatively modest yet maintaining an ascending order from internet-based, to friend-related, to family-related comparisons. Column 3 introduces the mediator into the baseline model. Both the treatment groups and stress remain significant, with stress correlating negatively with well-being—each unit increase in stress (*β* = −0.262, *p*-value = 0.000) is associated with 0.262 units decrease in well-being—supporting its role as a partial mediator. This result provides strong empirical support for the transactional model of stress (Lazarus et al., 1984), confirming that the cognitive appraisal of a comparative triggers stress response, which is a critical pathway through which social comparison impairs hedonic well-being. It is noteworthy that after incorporating stress, the coefficients for social comparison under different social distances retain their original ordinal pattern: friend comparisons continue to show the strongest association (*β* = −0.962, *p*-value = 0.000), followed by family (*β* = −0.629, *p*-value = 0.002), and internet-based comparisons (*β* = −0.603, *p*-value = 0.003).

**Table 4:** Mediation effect of stress.

	(1) Baseline	(2) Mediator model	(3) Full model
Dependent variable	Well-being	Stress in scenario	Well-being
Family group	−1.053*** (0.202)	1.617*** (0.251)	−0.629*** (0.198)
Friend group	−1.358*** (0.196)	1.510*** (0.244)	−0.962*** (0.192)
Internet group	−0.988*** (0.204)	1.467*** (0.254)	−0.603*** (0.199)
Stress in scenario (M)	Not included	Not included	−0.262*** (0.031)
Control variables	Yes	Yes	Yes
Observations	606	606	606
R-squared	0.228	0.230	0.310

The significance level is denoted: *** for *p* < 0.01. Control variables include age, age square, gender, ln(income), education, diverse hobbies, occupation, social engagement, life satisfaction, stress in daily life, relative well-being at income level, relative well-being at diet, health, self-esteem, seek help family, and seek help internet.

For further confirmation, we conduct a bootstrap test (1000 resamples) with bias correction. As shown in Table A-7, the Bonferroni-corrected 95% confidence intervals for indirect effects exclude zero across all comparison types, confirming the robustness of mediation effects. Standardized indirect effects range from −0.384 to −0.424, accounting for about one-third of the total effects based on bias-corrected ab/c ratios. These results suggest that social comparison correlates with reduced well-being alongside increased stress, with the mediated portion varying by social distance: the indirect effect is lowest for friend comparisons (29.1%), while family and internet comparisons each account for approximately 40% of the total association.

### 3.3 Subgroup analysis of help-seeking behavior

Our regression results provide strong empirical evidence that the source of seeking help is a key moderator of the relationship between wealth comparison and well-being, but its role is not uniformly protective. It is crucial that the context of social comparison and the sources of seeking help are aligned for support to be effective. These findings are consistent with the argument of Siedlecki et al. (2014) that supporting validity depends on context. We explain these heterogeneous effects through the distinct relational attributes of family, friends, and internet.

While families are the primary source of seeking help (*N* = 336), the strongest negative association with well-being was observed among those who sought help from family, corresponding to a reduction in well-being of 1.125 units (*β* = −1.125, *p*-value = 0.000). This effect is greater than that in the overall sample (*β* = −1.053, *p*-value = 0.000), with a difference of 0.072 units, suggesting upward wealth comparisons within families may trigger feelings of inadequacy, failure, or not meeting family expectations. In this sensitive context, initiating a help-seeking interaction may not be seen as receiving support, but rather as an admission of defeat to the closest relatives, exacerbating perceived burden and relationship anxiety (Tesser et al., 1988; Taylor et al., 2004), turning well-intentioned support mechanisms into a source of additional psychological stress and associated with reduced well-being (Lazarus et al., 1984) (Table 5).

**Table 5:** Subgroup analysis of seek help.

	(1) Overall	(2) Subgroup:Seek help family	(3) Subgroup: Seek help internet
Dependent variable: well-being			
Family group	−1.053*** (0.202)	−1.125*** (0.249)	0.385 (0.750)
Friend group	−1.358*** (0.196)	−1.019*** (0.249)	−1.901*** (0.646)
Internet group	−0.988*** (0.204)	−0.757*** (0.281)	−2.103*** (0.693)
Seek help family	−0.489*** (0.169)		
	
Seek help internet	−0.189 (0.245)		
	
Control variables	Yes	Yes	Yes
Observations	606	336	72
R-squared	0.228	0.257	0.378

The significance levels are denoted: *** for *p* < 0.01 and * for *p* < 0.1. Control variables include age, age square, gender, ln(income), education, diverse hobbies, occupation, social engagement, life satisfaction, stress in daily life, relative well-being at income level, relative well-being at diet, health, self-esteem. SUEST test (Holm-adjusted *p*-values): test the significant differences in regression coefficients among different sub samples; Holm-adjusted *p*-values: multiple testing correction to control the risk of false positives.

Friend-based wealth comparisons were consistently associated with well-being reduction across help-seeking groups, showing reductions of 1.019 and 1.901 units among those seeking help from family (*β* = −1.019, *p*-value = 0.000) and the internet (*β* = −1.901, *p*-value = 0.005), respectively. The difference between these coefficients was not significant (Table A-8), indicating a persistent effect. This underscores the potent and universal negative impact of peer comparisons, consistent with the local dominance effect (Zell and Alicke, 2010). These results underscore that wealth comparisons among friends—psychologically proximal—are associated with substantial well-being reduction and that help-seeking behavior does not appear to mitigate this association, highlighting the persistent influence of comparative dynamics among peers. The heterogeneous role of help-seeking behavior—sometimes exacerbating well-being loss—adds a crucial nuance to the stress-buffering hypothesis (Cohen and Wills, 1985).

Regarding wealth comparisons on the internet, the associated reduction in well-being was 2.103 units (*β* = −2.103, *p*-value = 0.004), which is the greatest. It suggests that the small subgroup that turns to the internet for help (*N* = 72) may represent a distinct population that is particularly vulnerable to the curated, unrealistic displays of wealth on social media. Their choice to seek help online might reflect a pre-existing lack of access to robust, high-quality offline support networks (family or friends), leaving them with fewer resources to buffer this potent stressor. Furthermore, in algorithm-driven platforms, the behavior of seeking help on internet may lead to further exposure to negative content, intensifying rather than alleviating the initial stress.

To formally test coefficient heterogeneity across subgroups, we employ Seemingly Unrelated Estimation (SUEST) with Holm–Bonferroni correction (Holm, 1979). As presented in Table A-8, the results indicate that the source of help-seeking significantly moderates the association between wealth comparisons and well-being, with particularly pronounced moderation for family and internet-based comparisons.

### 3.4 Drivers behind difference in well-being

This section will explore the drivers behind differences in well-being across different wealth gap contexts using multiple regression analysis. The outcome variable, differ_wellbeing, reflects an individual’s sensitivity (the magnitude of change in well-being) to upward wealth comparisons. We estimate the following model, given by Equation 2:


$$differ\_wellbeing=\delta Z_{i}+e_{i}$$ (2)

where differ_wellbeing is the difference in well-being between two scenarios, $Z_{i}\;$ is the vector of controls, δ is coefficient showing the relationship, $e_{i}$ is error term.

As shown in Column (1) of Table A-9, every unit increase in life satisfaction (0–10 scale, *β* = −0.429, *p*-value = 0.000) is associated with a reduction of 0.429 units in wealth comparison sensitivity, suggesting that individuals with higher intrinsic life fulfillment may be less influenced by relative economic standing. Similarly, a 10% increase in income (*β* = −0.402 for ln(income), *p*-value = 0.001) is associated with a decrease of 0.040 units in comparison sensitivity, our findings reveal that higher absolute income may serve as a buffer by reducing sensitivity to social comparisons. This aligns with the notion that higher absolute income provides a material and psychological buffer, reducing vulnerability to upward wealth comparisons (Cheung and Lucas, 2016; Clark et al., 2008). Moreover, each unit increase in the perceived importance of diet to well-being (*β* = 0.084, *p*-value = 0.035) is associated with a reduction of 0.084 units in comparison sensitivity, implying that advantages in basic living standards may partially mitigate the negative psychological effects of wealth comparisons.

Within the family comparison group, the negative association between life satisfaction (*β* = −0.539, *p*-value = 0.000) and comparison sensitivity is larger than in the overall model, with a difference of 0.110 units, suggesting that comparisons with family members may intensify the moderating role of life satisfaction. Age (*β* = −0.187, *p*-value = 0.091) shows a positive association with comparison sensitivity—each additional year of age is linked to an increase of 0.187 units in the well-being difference, possibly reflecting older individuals’ heightened sensitivity to economic norms within the family (e.g., childcare and eldercare expenses) (Lachman et al., 2015). Paradoxically, every unit increase in family harmony (0–10 scale, *β* = −0.328, *p*-value = 0.006) is associated with an increase of 0.328 units in comparison sensitivity, implying that pressures to maintain familial harmony may exacerbate psychological strain, which is associated with wider well-being disparities.

In the friend comparison group, the negative association of life satisfaction (*β* = −0.357, *p*-value = 0.000) is smaller than in the overall group by 0.072 units. A 10% increase in income (*β* = −0.625 for ln(income), *p*-value = 0.002) is associated with a reduction in comparison sensitivity of 0.063 units and this effect is larger than the overall group, underscoring the “visibility effect” of income comparisons among peers, where relative economic standing is more readily observable and salient (Cheung and Lucas, 2016). Additionally, sharing birthdays—a binary variable indicating whether to remember and celebrate friends’ birthday (if yes equals 1)—is negatively associated with comparison sensitivity (*β* = −1.389, *p*-value = 0.013), corresponding to a decrease of 1.389 units. From the perspective of construal-level theory (Trope and Liberman, 2010), rituals such as birthday celebrations reduce psychological distance between friends, prompting a shift from abstract comparisons of wealth to concrete experiences of intimacy, thereby reducing sensitivity to wealth disparities.

In the internet-based comparison group, relative to the overall model, the negative association of life satisfaction (*β* = −0.366, *p*-value = 0.000) is smaller by 0.063 units. A 10% increase in income (*β* = −0.457 for ln(income), *p*-value = 0.078) is associated with a decrease of 0.046 units in comparison sensitivity. Education—a binary variable indicating the level of education (if the education is a bachelor’s degree or above, equals 1)—exhibits a notable negative association (*β* = −0.637, *p*-value = 0.083), indicating that highly educated individuals demonstrate comparison sensitivity that is 0.637 units lower, on average, than those with lower education. This implies that greater education may enhance individuals’ ability to discern the curated nature of online self-presentation, which is associated with a reduction in irrational comparisons. Furthermore, the positive association of diet-related well-being (*β* = 0.190, *p*-value = 0.019) is larger than in the overall group by 0.106 units, emphasizing how non-economic elements—such as lifestyle displays that is frequently symbolic and highly visible on social media—can alter comparison processes in digital spaces.

Overall, these findings confirm that life satisfaction and absolute income are consistently associated with reduced comparison sensitivity. As a core component of subjective well-being (Lyubomirsky, 2001), life satisfaction serves as an internal buffer, while absolute income provides an external buffer against the negative effects of relative deprivation (Cheung and Lucas, 2016; Oishi et al., 2022). Notably, previous research shows that relative income is a stronger predictor of life satisfaction than absolute income (Cheung and Lucas, 2016; Clark et al., 2008). Moreover, the context-specific drivers (e.g., family harmony, friends’ birthdays) further enrich our understanding by highlighting how relational dynamics can paradoxically intensify or mitigate comparison sensitivity within different social spheres (Sirgy, 2019).

## 4 Conclusion

This study investigates the association between wealth comparison and reduced well-being across social distance by employing questionnaire-based data collection and regression analysis. The most pronounced adverse effects were observed within friend groups, compared to family and internet groups. Stress partially mediates this relationship, while help-seeking behaviors exhibit heterogeneous moderating effects. Additionally, life satisfaction and income emerge as critical determinants of well-being disparities across these contexts.

The results, discussed in the context of existing theoretical frameworks, can be attributed to different social dynamics. In the family, the pressure to maintain superficial harmony may exacerbate psychological stress. Among friends, greater income disparities are associated with exacerbate feelings of relative deprecation, while stronger emotional bonds are correlated with improved well-being. The existence of the network promotes upward comparisons and individuals with higher education appear better equipped to recognize this selectivity, potentially mitigating some irrational comparison effects.

Based on these findings, we provide some recommendations: For individuals, the first step is to recommend actively regulate social behavior, including increasing communication and organizing emotional connection activities (e.g., birthday celebrations). Second, people need to respond rationally to social media and avoid blind comparisons. Third, we encourage everyone to develop hobbies and reduce dependence on external evaluations. For the government, first, it is recommended to promote authentic and diverse content representation on social media, encouraging platforms to highlight ordinary life and non-material values rather than fostering materialistic competition and particular attention could be directed to vulnerable groups such as adolescents. The second is to promote public mental health support, such as incorporating psychological education into public publicity, improving public awareness and coping ability. The third is to improve the economic safety net, strengthen unemployment benefits, income subsidies and other security policies, and alleviate people’s financial anxiety.

This study has several limitations. First, this study is not designed to establish causality. Second, the high proportion of young, highly educated individuals, limiting the generalizability of the findings to broader populations. Third, it should be noted that our measure of help-seeking behavior captured a binary preference for the source of support but did not assess the frequency, quality, or perceived effectiveness of the support received and the limited size of the internet-based help-seeking subgroup may constrain the statistical power and generalizability of the corresponding findings. Future research should expand the timeframe and enhance sample diversity to improve the context-specificity of interventions and employ more nuanced measures to explore how different dimensions influence socially comparable well-being outcomes.

## Data Availability

The original contributions presented in the study are included in the article/Supplementary material, further inquiries can be directed to the corresponding author.
