# Supplementary material for: Wealth comparison across social distances: implications for well-being
Source: Front Psychol. 2025 Nov 26;16:1661009. doi: 10.3389/fpsyg.2025.1661009 (PMC12689371; doi:10.3389/fpsyg.2025.1661009)
Supplement: Supplementary file 1 [file Table_1.pdf]

Appendix

Table A-1 Question in Scenarios

Variable	Question (After learning about his/her richer living conditions, ...)	Scale
Well-being	..., your well-being is...	0-10
Satisfaction	..., your satisfaction is...	0-10
Stress in scenario	..., what is the stress you feel in this scenario?	0-10
Meaning	..., how much meaning do you think this kind of life is?	0-10
Change	..., what is your willingness to change the status quo in your life?	0-10
Reality	..., how much do you think this life is true?	0-10

Table A-2 Variable Definition and Measurement Scales

Variable	Definition/Measurement	Unit/Scale
Overall		
Age	age of the participant	Years
Age square	age*age	Squared years
Gender	male=1, female=0	Binary (0/1)
Income	income per year	¥10,000
Education	bachelor' degree or above=1, below bachelor=0	Binary (0/1)
Occupation	student=0, worker=1, business/service=2, others=3	Categorical (0-3)
Diverse hobbies	have more than three different hobbies=1	Binary (0/1)
Social engagement	active participation=10, no participation=0	Scale (0-10)
Life satisfaction	very satisfied=10, completely dissatisfied=0	Scale (0-10)
Stress in daily life	seriously stress=10, no pressure=0	Scale (0-10)
Relative well-being at income level	association of income level on well-being	Scale (0-10)
Relative well-being at diet	association of diet on well-being	Scale (0-10)
Self-esteem	importance of self-esteem	Scale (0-10)
Health	have chronic diseases or mental disorders=1	Binary (0/1)
Seek help family	prefer to seek help from family=1	Binary (0/1)
Seek help internet	prefer to seek help from internet=1	Binary (0/1)
Family group		
Living with family	living with family=1	Binary (0/1)
Family gatherings and trips	frequency per year more than three=1	Binary (0/1)
Communication with family	high frequency=1	Binary (0/1)
Family harmony	vary harmonious=10, not harmonious=0	Scale (0-10)
Friend group		
Close friends	number of close friends	Continuous
Ideal number of friends	number of close friends in an ideal state	Unit
Offline gatherings	more than three times=1	Binary (0/1)
Friends' birthday	remember and celebrate friends' birthday=1	Binary (0/1)

Internet group		
Electronic devices	frequency per day more than 8 hours=1	Binary (0/1)
Social media	frequency per day more than 2 hours=1	Binary (0/1)
Purpose	main purpose is study or work=1, entertainment=0	Binary (0/1)
Social media interaction	high frequency=1	Binary (0/1)
Credibility	credibility of social media information	Scale (0-10)

Table A-3 Common Method Bias

Factor	Eigenvalue	Difference	Proportion	Cumulative
Factor 1	3.40006	1.65194	0.1790	0.1790
Factor 2	1.74812	0.05803	0.0920	0.2710
Factor 3	1.69009	0.06575	0.0890	0.3599
Factor 4	1.62434	0.04614	0.0855	0.4454
Factor 5	1.57820	0.37174	0.0831	0.5285
Factor 6	1.20646	0.15431	0.0635	0.5920
Factor 7	1.05214	0.09833	0.0554	0.6473

Note: Extraction Method: Principal Component Analysis. The first factor accounted for 17.83% of the variance, which is below the 50% threshold, indicating that common method bias is not a significant concern.

Table A-4 Summary Statistics in Different Groups

Variable	Mean	Standard Deviation	Minimum	Maximum
Panel A: Family group (N=100)				
Age	28.727	9.825	18	56
Gender	0.515	0.500	0	1
Education	0.869	0.336	0	1
Income	10.047	10.074	1.2	60
Occupation	1.242	1.190	0	3
Health	0.222	0.421	0	1
Diverse hobbies	0.859	0.367	0	1
Social engagement	6.505	1.836	2	10
Life satisfaction	7.222	1.768	1	10
Stress in daily life	5.758	2.179	1	10
Self-esteem	7.626	1.632	1	10
Relative well-being at income level	7.606	1.803	1	10
Relative well-being at diet	8.091	1.720	1	10
Seek help family (D)	0.620	0.488	0	1
Seek help internet (D)	0.100	0.302	0	1
Living with family	0.740	0.439	0	1
Family gatherings and trips	0.780	0.414	0	1
Communication with family	0.850	0.357	0	1
Family harmony	7.990	1.396	1	10
Panel B: Friend group (N=97)				

Age	28.453	11.167	17	65
Gender	0.491	0.500	0	1
Education	0.877	0.328	0	1
Income	8.149	10.788	1	100
Occupation	1.132	1.150	0	3
Health	0.160	0.367	0	1
Hobbies	0.877	0.305	0	1
Social engagement	6.774	1.873	2	10
Life satisfaction	7.453	1.646	3	10
Stress in daily life	6.255	2.512	0	10
Self-esteem	7.604	1.787	1	10
Relative well-being at income level	7.047	1.936	0	10
Relative well-being at diet	8.000	2.106	1	10
Seek help family (D)	0.575	0.497	0	1
Seek help internet (D)	0.132	0.340	0	1
Close friends	3.302	1.849	0	9
Ideal number of friends	4.311	2.721	0	15
Offline gatherings	0.736	0.441	0	1
Friends' birthday	0.925	0.264	0	1
Panel C: Internet group (N=106)				
Age	27.134	8.834	18	54
Gender	0.495	0.500	0	1
Education	0.814	0.397	0	1
Income	8.129	9.693	1	60
Occupation	0.959	1.108	0	3
Health	0.103	0.304	0	1
Hobbies	0.866	0.351	0	1
Social engagement	6.742	1.609	2	10
Life satisfaction	7.340	1.776	2	10
Stress in daily life	6.320	2.636	0	10
Self-esteem	7.866	1.903	1	10
Relative well-being at income level	6.835	1.332	2	10
Relative well-being at diet	8.433	1.960	0	10
Seek help family (D)	0.464	0.501	0	1
Seek help internet (D)	0.124	0.331	0	1
Electronic devices	0.515	0.500	0	1
Social media	0.773	0.419	0	1
Purpose	0.330	0.470	0	1
Social media interaction	0.361	0.480	0	1
Credibility	6.000	1.499	2	9

Note: Dummy variables are denoted as D in parentheses.

Table A-5 Correlation Coefficient of Key Variables

	Well-being	Family group	Friend group	Internet group	Stress in daily life	Seek help friend	Seek help internet	Satisfaction
Well-being	1.000							
Family group	-0.102	1.000						
Friend group	-0.191	-0.205	1.000					
Internet group	-0.100	-0.194	-0.201	1.000				

Stress in daily life	-0.362	0.150	0.145	0.130	1.000			
Seek help friend	-0.130	-0.044	-0.034	0.080	0.072	1.000		
Seek help internet	-0.111	-0.026	0.019	0.007	0.036	-0.256	1.000	
Satisfaction	0.670	-0.247	-0.277	-0.132	-0.132	-0.055	-0.082	1.000

Table A-6 VIF Test Results

Variable	VIF	1/VIF
Age	66.25	0.015093
Age square	56.69	0.017640
Occupation	2.50	0.400610
Ln(income)	1.75	0.570336
Social engagement	1.59	0.629171
Seek help family	1.43	0.696974
Life satisfaction	1.43	0.697911
Stress in daily life	1.34	0.746321
Seek help internet	1.27	0.785492
Relative well-being at diet	1.24	0.804411
Education	1.24	0.809318
Health	1.18	0.845783
Gender	1.17	0.852654
Relative well-being at income level	1.17	0.857133
Diverse hobbies	1.17	0.858296
Family group	1.14	0.878676
Internet group	1.13	0.881405
Friend group	1.13	0.888063
Self-esteem	1.12	0.893397
Mean VIF	7.67	

Table A-7 Mediator Effect Test of Stress

Variable	Indirect effect	Boot SE	95%CI (P)	95%CI (BC)
Family group	-0.424***	0.086	[-0.599, -0.266]	[-0.614, -0.279]
Friend group	-0.396***	0.093	[-0.581, -0.219]	[-0.594, -0.231]
Internet group	-0.384***	0.094	[-0.575, -0.219]	[-0.586, -0.229]

Notes: The significance level is denoted: *** for $p < 0.01$.

Table A-8 Chi-square Test for Subgroup Analysis of Seek Help

	chi-square	df	P-value
Family group	5.35	1	0.0622*
Friend group	1.71	1	0.1914*
Internet group	4.55	1	0.0660*
All	12.01	3	0.0073

Note: The SUEST (Seemingly Unrelated Estimation) test examines whether regression coefficients differ significantly across subgroups; P-values marked with * are Holm-adjusted to control the risk of false positives.

Table A-9 Drivers behind Difference in Well-being

Dependent variable: differ_well-being

	(1) Overall	(2) Family group	(3) Friend group	(4) Internet group
Life satisfaction	-0.429***	-0.539***	-0.357***	-0.366***
	(0.049)	(0.088)	(0.097)	(0.096)
Ln(income)	-0.402***	-0.343	-0.625***	-0.457*
	(0.122)	(0.226)	(0.203)	(0.258)
Relative well-being at diet	0.084**	0.113	0.066	0.190**
	(0.040)	(0.079)	(0.079)	(0.080)
Family harmony		0.328***		
		(0.118)		
Friends' birthday			-1.389**	
			(0.551)	
Age	0.075	0.187*	0.019	0.016
	(0.057)	(0.110)	(0.090)	(0.166)
Education	-0.096	0.043	0.166	-0.637*
	(0.220)	(0.456)	(0.432)	(0.366)
Control variables	Yes	Yes	Yes	Yes
Observations	606	200	212	194
R-squared	0.187	0.281	0.208	0.247

Notes: The significance levels are denoted: *** for $p < 0.01$, ** for $p < 0.05$, and * for $p < 0.1$. Control variables include age square, diverse hobbies, occupation, social engagement, relative well-being at income level, self-esteem, stress in daily life, seek help family, seek help internet, living with family, family gatherings and trips, communication with family, number of close friends, number of close friends in an ideal state, offline gatherings of friends, friends' birthday, electronic devices, social media, purpose of social media use, social media interaction, credibility of social media information.
